# Effective transition from standing to groundwork combat: an analysis of judo athletes with visual impairments

**DOI:** 10.3389/fspor.2025.1729837

**Published:** 2026-01-09

**Authors:** Daniele Detanico, Vinícius de Oliveira Souza Gulias, Nathalie Azeredo Bahiense Gomes, Rafael Lima Kons

**Affiliations:** 1 Biomechanics Laboratory, Center of Sports, Federal University of Santa Catarina, Florianópolis, Brazil; 2 Department of Physical Education, Faculty of Education, Federal University of Bahia, Salvador, Brazil

**Keywords:** combat sports, competitive performance, para athletes, skills, tactical strategies

## Abstract

Judo for athletes with visual impairments (VI judo) requires constant adaptation of technical and strategic skills. The transition from standing to groundwork combat (*tachi-waza* to *ne-waza*) is a key phase that impacts match outcomes. VI judo athletes are classified into two groups: J1 (total blindness) and J2 (partially sighted), each facing distinct challenges. This study examined the effective transitions (those that resulted in scoring actions) from standing to groundwork combat in high-level VI judo athletes considering J1 and J2 sport classes. In this observational study, a total of 195 videos was analyzed, involving 146 VI judo athletes who competed at the Paris 2024 Paralympic Games. Chi-square tests were used to verify the associations between effective actions and transition outcomes, with significance level set at *p* < 0.05. Main results showed that J1 athletes had a higher frequency of effective transitions compared to J2 athletes [*χ*² = 4.81; *p* = 0.028 (medium effect)], with *osae-komi-waza* (immobilization techniques) being the most used technique across both groups [*χ*² = 11.24; *p* = 0.004 (large effect)] and results in ippon [*χ*² = 6.80; *p* = 0.008 (medium effect)]. No significant differences were found between J1 and J2 athletes regarding transition pace, *uke* (athlete who receives the technique) body position and match status (*p* > 0.05). The results suggest that J1 athletes experienced higher frequency of effective transitions from standing to groundwork combat compared to J2 athletes considering *osae-komi-waza* techniques which results in ippon more frequently for both groups. These findings provide preliminary evidence on the performance characteristics of VI judo athletes, particularly regarding effective transitions to groundwork techniques. They also supply technical-tactical information that can support athlete development across sport classes under the new classification system.

## 1 Introduction

Judo for athletes with visual impairments (VI judo) is a highly dynamic sport and, similar to conventional judo, requires constant adaptation of technical and strategic skills (1). It involves intermittent movements, varying gripping techniques (*kumi-kata*), intensity fluctuations, and transitions between phases such as attack, groundwork (*ne-waza*), and pauses (2, 3). Judo functions as a system in which athletes alternate between controlling and responding to their opponent's actions, creating a cyclical pace of effort and rest (4). However, in VI judo the lack of vision introduces specific challenges that require athletes to adapt (5). For example, matches begin with the grip on the *judogi*, and the combat is constantly assisted and restarted by the referee (6).

Athletes are classified into two distinct groups based on their level of visual impairment: J1 (total blindness) and J2 (partially sighted) (7, 8). This classification is determined by a combination of visual acuity and visual field measurements (5). These distinctions significantly influence the strategies and techniques that athletes use during competition, emphasizing the need for a specialized approach to the technical-tactical analysis of judo for athletes with different levels of visual impairment (7–10). In this context, exploring competition-related performance provides a valuable approach, especially by quantifying the specific actions of athletes from different sports classes in official competitions. For example, a recent study carried out by Kons et al. (11) reported significant differences in performance indices (e.g., score index and technical variability in the groundwork combat) between J2 and J1 athletes, highlighting distinct characteristics to each group.

With regard to performance of VI judo in official competition over the last few years there has been an increasing interest in analyzing the performance of VI judo matches, providing detailed insights into the time-motion dynamics of actions (12–14), most frequently executed techniques (15), performance variability analysis and its correlation with success (1, 11), contextual factors influencing performance (6), and analysis of grip situations (16). These studies emphasize the importance of identifying key performance aspects in VI judo, considering factors such as sport classes (i.e., levels of visual impairment), sex, weight categories and competitive outcomes. In this context, a recent study by Gutierrez-Santiago et al. (17) analyzed 232 combats and 313 scoring actions at the 2018 World Championships and found that the highest scoring frequency occurred within the first two minutes, with *ashi-waza*, *te-waza*, and *sutemi-waza*, particularly *sumi-otoshi*, *ouchi-gari*, and *ko-soto-gake*, being the most effective techniques. Lapel–sleeve grip was the most prevalent and successful, considering weight categories. Finally, the authors highlight that lighter athletes commonly scored *waza-ari* by countering leg attacks with arm or sacrifice techniques, whereas heavier athletes frequently achieved *ippon* through leg-to-leg counterattacks, overall identifying early initiative, grip efficiency, and effective standing-to-groundwork transitions as key determinants of performance.

A key aspect in the performance in official competitions, which has recently been highlighted in able-bodied judo, is the transition from standing to groundwork combat, showing important impact on match outcomes (2, 18–21). The transition from *tachi-waza* (standing fight) to *ne-waza* (ground fight) can occur in several scenarios: when an athlete lands without scoring or after a *waza-ari* (half score), allowing either athlete to continue in *ne-waza*; when one athlete skillfully takes their opponent to the ground without executing a throwing technique; or when an athlete falls or is about to fall, giving the opponent the opportunity to transition into *ne-waza*. These transitions are crucial for contest outcomes (18). As an example, in able-bodied athletes a traditional study conducted by Rox et al. (21) reported that transitions to the groundwork matches most often occurred after the opponent blocked an attack (50%), after an escape (28%), following a throw (18%), and after a counter-attack (2%). Despite these opportunities, 42% of situations deemed favorable for continuing into *ne-waza* were not exploited. In another study, Pierantozzi et al. (19) reported that only 27% of transition opportunities led to a *ne-waza* sequence, even though there were an average of eight transitions per match. Similarly, Pierantozzi et al. (20) demonstrated that 7% of transitions resulted in *ippon*, and 11% resulted in *waza-ari*. These findings highlight that the transition phase is critical for determining the contest outcome. More recently, Nagai et al. (2) analyzed the transition to the groundwork and reported that 75% of scoring actions in junior and senior high-level able-bodied judo athletes were achieved through *osae-komi-waza*, with the majority (71%) occurring when the match was tied.

Given the limited evidence on athlete performance during transitions from standing to groundwork, particularly transitions that successfully led to scoring actions (e.g., effective transitions), and considering the new classification system that divides VI judo athletes into two groups based on impairment severity, characterizing these effective transitions for both groups is essential. This analysis will focus on the classifications proposed by Nagai et al. (2), which define performance differences based on the complexities of judo (e.g., pace of transition and technique type). Thus, this study aimed to: (a) examine effective transition from standing to groundwork combat in high-level VI judo athletes who competed at the Paris 2024 Paralympic Games; (b) compare the pace of transition, technique type, moment (e.g., minute of occurrence), status, and scores between J1 and J2 groups. Our primary hypothesis is that the *osae-komi-waza* (immobilization techniques) will be the most frequently used, as these are common in VI judo (15), with no significant differences between J1 and J2 due to the similarities in their transition situations.

## 2 Methods

### 2.1 Design

This study can be considered cross-sectional in a general sense, as it analyzes a single competition; however, from an observational methodology perspective, it follows a nomothetic, follow-up (longitudinal), and multidimensional design, as recommended by Anguera et al. (22) for analyses of high-level judo athletes with visual impairments competing at the Paris 2024 Paralympic Games. Analyses were conducted for each sport class (J1 and J2) and considered sex, weight category, competition phase, pace of transition, type of technique, uke body position, match moment, status, and scores. Observational methodology provides a systematic yet flexible framework for examining naturally occurring events in judo matches. Accordingly, the study adopted a nomothetic design by analyzing effective transitions from standing to groundwork across all VI judo athletes, a longitudinal approach by evaluating behavioral consistency across multiple matches during the Paris 2024 Games, and a multidimensional perspective incorporating the various criteria defined in the observational instrument, following procedures similar to those described by Gutiérrez-Santiago et al. (17).

### 2.2 Participants

A total of 146 VI judo athletes participated, including 78 males and 68 females. Of these, 78 were classified as J1 (totally blind) and 68 as J2 (partially sighted), distributed across four weight categories for both male and female VI judo. Additional athlete information was obtained from the official results book of the Paris 2024 Paralympic Games (https://ibsajudo.sport/wp-content/uploads/2024/10/Judo-Result-Book.pdf).

### 2.3 Match analysis and outcomes

Two expert researchers, each with over 10 years of judo experience, completed the video analysis protocol for each match. Analysis was conducted of all matches from 146 VI judo athletes at the Paris 2024 Paralympic Games using official videos (*n* = 195 matches) from the International Paralympic Committee (IPC), available on their YouTube channel (https://www.youtube.com/user/ParalympicSportTV). The primary focus was to identify matches where transitions resulting in scores occurred through the application of *katame-waza* (grappling techniques such as *osae-komi-waza*, *shime-waza*, or *kansetsu-waza*). After identifying these occurrences, each sequence leading to a score was analyzed to determine the actions involved. To reduce errors related to technique types and scores, the Paris 2024 Paralympic Games results book (https://ibsajudo.sport/wp-content/uploads/2024/10/Judo-Result-Book.pdf) was consulted for more accurate confirmation of the results. In this study, observers first participated in a structured training session for video analysis, during which they reviewed examples of transitions and discussed the operational definitions for each category in the spreadsheet. Following this training, all matches were entered and processed independently in the spreadsheet by each observer, after which the spreadsheet data were compared in a consensual verification process. Any discrepancies identified were resolved through joint discussion until full agreement was reached, ensuring consistency and reliability in the recorded data.

A spreadsheet was created in Excel for data collection, which included the following columns: (1) sex (male, female); (2) weight category (extra-lightweight, lightweight, middleweight and heavyweight); (3) sport class (J1 = total blind, J2 = partially sighted); (4) competition phase (eliminatory, quarterfinals, semifinals, repechage, bronze, finals); (5) transition pace, which indicated whether the athlete performed the groundwork technique immediately after the throw, via a consecutive link, or progressed on the ground. The definitions used were as follows: immediate transitions (control maintained with no loss within 5 s, especially for *shime-waza* or *kansetsu-waza*); consecutive transitions (slight loss of control but recovery within 5–7 s to secure *ne-waza*); and progressive transitions (control achieved after more than 7 s, involving contested ground movements). Column (6) recorded the technique type (*osae-komi-waza*, *kansetsu-waza*, or *shime-waza*); (7) Uke body position during the transition was recorded to characterize the defensive posture adopted by the athlete receiving the technique at the moment of transition from standing to groundwork combat. Positions were categorized based on standardized judo terminology as follows: turtle position, defined as the uke adopting a compact posture with knees and elbows on the mat and the trunk flexed to protect against attacks; supine position, when the uke was lying on the back with the torso facing upward; prone position, when the uke was lying face down on the mat; guard position, characterized by the uke controlling the opponent with the legs while positioned on the back; and seated position, when the uke was sitting on the mat with partial trunk elevation; (8) noted the combat status (tied, tori winning, or tori losing at the transition moment); and (9) indicated the fight moment in minutes [<1 min, 1–2 min, 2–3 min, 3–4 min, golden score (>4 min)]; (J) score attribution (*e.g ippon* or *wazari*). The observations were recorded in an Excel spreadsheet specifically developed for this study. Both experts involved have at least five years of experience analyzing videos and tabulating data in Excel. This approach to data handling adheres to the ethical principles of the Belmont Report (23), ensuring that participants' autonomy and confidentiality are maintained.

### 2.4 Statistical analysis

The normality of variable distributions was assessed using the Kolmogorov–Smirnov test. The distribution of dependent variables was assessed, and percentages for each were presented in relation to the independent variables. Chi-square (*χ*²) tests were used to determine any associations between technique actions and factors such as sex, sport classes, weight categories and competition phase, followed by Cramer's *V* effect size for the key comparisons. A Cramer's *V* value greater than 0.20 indicates a small effect, a value from 0.21–0.35 suggests a medium effect, and a value above 0.35 signifies a large effect, with Chi-square tests having two degrees of freedom (24). The intra- and inter-rater reliability were assessed using the Kappa coefficient prior to the main data analysis, based on matches from a different competition to avoid bias from the study sample. The Kappa coefficients were calculated globally across all variables, providing an overall measure of inter-observer agreement. A total of twelve videos was used to calculate reliability of the analysis. Statistical significance was set at *p* < 0.05 and all analyses were performed using JASP software (version 0.11.1, JASP Team, University of Amsterdam, Netherlands).

## 3 Results

The intra and inter-rater reliability were assessed using Kappa coefficient, following the guidelines of Landis and Koch (25). Across all variables analyzed in the study and for both raters, kappa values ranged from 0.90 to 0.95, indicating near-perfect agreement. Seventy-six effective transitions from standing to the groundwork combat were identified during the Paris 2024 Paralympic Games, with 47 (62.7%) from the J1 group and 28 (37.3%) from the J2 group. The distribution revealed significant differences in effective transitions to the groundwork between two groups [*χ*² = 4.81; *p* = 0.028; Cramer's *V* = 0.35 (medium effect)], with a higher frequency observed in the J1 group. In Table 1 is showed the association between the frequency of effective transitions to groundwork matches in judo athletes with visual impairments, analyzed by sex, weight category, and competition phase for the J1 and J2 groups. No significant associations were found across all groups (*p* > 0.05). These results suggest that athletes with total visual impairment (J1) may rely more on strategic transitions to groundwork.

**Table 1:** Absolutely and relative frequency of effective transition from standing to groundwork combat in VI judo athletes according to sex, weight category and competition phase in J1 and J2 groups.

Groups	J1	J2	*χ*²	*p*	Cramer's *V*
	*n* (%)	*n* (%)			
Sex					
Male	22 (62.8)	15 (37.5)	0.01	0.97	0.04
Female	25 (62.5)	13 (37.1)			
Weight category					
EL	15 (68.1)	7 (31.8)	4.89	0.17	0.25
LW	10 (52.6)	9 (47.3)			
MW	15 (78.9)	4 (21.0)			
HW	7 (46.6)	8 (53.3)			
Competition phase					
Eliminatory	8 (80.0)	2 (20.0)	3.55	0.61	0.21
Quarterfinals	14 (63.6)	8 (36.3)			
Semifinals	10 (62.5)	6 (37.5)			
Repechage	6 (60.0)	4 (40.0)			
Bronze	3 (37.5)	5 (62.5)			
Finals	6 (66.6)	3 (33.3)			

EL, extra-lightweight; LW, lightweight; MW, middleweight; HW, heavyweight.

Table 2 shows the association between the frequency of effective transitions from standing to groundwork combat in judo athletes with visual impairments, analyzed by pace of transition, type of judo technique and *uke* body position for J1 and J2 groups. Significant association was detected only for type of judo technique, with more frequency for *osae-komi-waza* for both groups (large effects). No association was found from J1 and J2 groups and pace of transition and *uke* body position.

**Table 2:** Absolute and relative frequency of effective transition from standing to groundwork combat in VI judo athletes according to pace of transition, type of judo technique and *uke* body position for J1 and J2 groups.

Groups	J1	J2	χ²	*p*	Cramer's *V*
	*n* (%)	*n* (%)			
Pace of transition					
Immediate	31 (68.8)	14 (31.1)	3.85	0.14	0.22
Consecutive	2 (100.0)	0 (0.0)			
Progressive	14 (50.0)	14 (50.0)			
Type of technique					
*Osae-komi-waza*	41 (69.4)	18 (30.5)	**11** **.** **27**	**0** **.** **004**	**0** **.** **38**
*Kansetsu-waza*	6 (60.0)	4 (40.0)			
*Shime-waza*	0 (0.0)	6 (100.0)			
Uke body position					
Turtle	10 (71.4)	4 (28.5)	2.42	0.65	0.18
Supine	18 (60.0)	12 (40.0)			
Prone	14 (70.0)	6 (30.0)			
Half-guard	1 (50.0)	1 (50.0)			
Seated	4 (44.4)	5 (55.5)			

**Bold** = significant association.

Table 3 presents the association between the frequency of effective transitions from standing to groundwork combat in judo athletes with visual impairments, analyzed by match moment (minute of occurrence), status, and scores for the J1 and J2 groups. A significant association was found for scores (medium effect). No significant associations were observed for the other variables across all groups (*p* > 0.05).

**Table 3:** Absolute and relative frequency of effective transition from standing to groundwork combat in VI judo athletes according to moment, status and scores for J1 and J2 groups.

Groups	J1	J2	χ²	*p*	Cramer's *V*
	*n* (%)	*n* (%)			
Moment					
<1	13 (54.1)	15 (65.2)	1.53	0.82	0.14
1–2	15 (62.2)	8 (34.7)			
2–3	7 (63.6)	4 (36.3)			
3–4	8 (66.6)	4 (33.3)			
>4	4 (80.0)	1 (20.0)			
Status					
Tied	25 (67.5)	12 (32.4)	0.86	0.64	0.10
Winning	20 (58.8)	14 (41.1)			
Losing	2 (50.0)	2 (50.0)			
Scores					
Ippon	44 (69.8)	19 (30.2)	6.8	0.008	0.30
Wazari	3 (25.0)	9 (75.0)			

## 4 Discussion

This study examined successful transition phases from standing to groundwork combat in high-level VI judo athletes who competed at the Paris 2024 Paralympic Games. The hypothesis of this study was confirmed, as the *osae-komi-waza* (immobilization techniques) was the most frequently used technique in both J1 and J2 groups, and the transition situations were similar across the groups.

The J1 group exhibited a higher frequency of effective transitions from standing to groundwork combat compared to J2 group. This difference may be attributed to the fact that J1 athletes who have higher level of visual impairment tend to use more frequently groundwork techniques (7, 15). Groundwork techniques provide athletes greater control over their opponents due to the close-contact nature, which is often intense (7, 18). No significant differences were observed between J1 and J2 groups in terms of sex, weight category or competition phase, indicating that the effective of transitions to the groundwork was similar across all variables, including male and female athletes, the four weight categories, and different competition phases. Notably, with the recent classification change, J1 and J2 athletes now compete separately based on the degree of impairment (5), and weight categories (reduced from seven to four) can influence this behavior (7). The variations observed across weight categories highlight an important consideration in light of recent changes, particularly for athletes who had to adapt to a higher or lower category (7), necessitating adjustments to technical-tactical strategies to accommodate the wider weight range (7, 8). Despite these changes, the performance profiles of athletes in both groups appear similar (6).

*Osae-komi-waza* was the group of technique most frequently used by athletes, following an effective transition to groundwork combat in VI judo. In other words, immobilizations are preferred techniques by both J1 and J2 athletes over other *ne-waza* techniques, such as *kansetsu-waza* and *shime-waza*. Kons et al. (15) found a higher prevalence of *osae-komi-waza* techniques (40%–60%) among VI athletes, regardless of sport class, suggesting that athletes with visual impairments tend to favor techniques that provide greater control of the opponent, following an effective transition to groundwork combat. In line with this, Nagai et al. (2) observed a higher frequency of *osae-komi-waza* techniques among able-bodied judo athletes, with senior male athletes (69.7%), senior female athletes (79%), junior male athletes (76%), and junior female athletes (74%) demonstrating a similar pattern. This suggests a comparable preference for this type of technique across different categories of judo athletes. Another important aspect is that *shime-waza* techniques were predominant among VI judo athletes in the J2 group. This may be explained by their greater visual acuity/field (27), which facilitates transitions and execution of these more complex and controlled techniques, particularly when performed from standing (15, 17).

No significant association was found between the pace of transition and the *uke* body position, suggesting that these factors are not influenced by the visual impairment of judo athletes regardless of sport class (J1 and J2). For instance, able-bodied judo athletes exhibited a higher progressive pace of transition (34% for female and 28.3% for male) compared to junior athletes (21% for female and 16% for male) (2). This difference may be attributed to senior athletes' greater experience, allowing them to perform more progressive actions within their visual field (26). In contrast, athletes with visual impairments show few progressive transitions, likely due to reduced acuity and a narrower visual field (1, 5, 27).

The moment of the match, status, and scores did not significantly differ between VI judo athletes in the J1 and J2 classes. The results point to a possible similarity between the two sport classes, suggesting that effective transitions to groundwork combat could be a common aspect of high-level competition for both J1 and J2 athletes (6, 15). This supports the notion that, despite differences in visual impairment, both groups exhibit comparable strategies and execution during this match phase, where the highest number of transitions result in an ippon for both classes (11). In contrast, Nagai et al. (2) found that 81% of the transitions from standing to groundwork combat in senior able-bodied female judo athletes' matches ended in a tie at some point, underscoring the unique competitive dynamics of that group and their specific performance strategies. It is also important to note that the VI judo classification system was recently modified, and Paris 2024 is the first Paralympic Games held under this new system. As a result, VI judo athletes may still be adapting technical-tactical strategies, considering the changes in the visual classification system.

This research contributes to understanding the transition phases in high-level VI judo competitions, there are several limitations to consider. First, the study was conducted within the context of the Paris 2024 Paralympic Games, which is the first Paralympic Games held under the new VI judo classification system (28). In this sense, athletes are still adapting to the updated system, and possibly their tactical strategies and techniques may not yet fully reflect their potential under the new sport class groups. Additionally, the study primarily focuses on comparing the J1 and J2 classes, which are defined by the degree of visual impairment, and athletes now compete separately according to the new classification (Krabben et al., 2020). This distinction significantly reduced the sample size, with only seventy-six occurrences identified in the video analysis of the matches. Finally, given the specificity of the group analyzed, the sample is considered adequate for the purposes of this study. Nevertheless, some categories (e.g., *shime-waza*, consecutive transitions, certain uke positions) had very low frequencies, which may compromise the stability of Chi-Square tests and should be interpreted with caution. Although the sample may appear limited, it reflects the relatively small size of the VI judo population, with only 146 athletes participating at the Paris 2024 Paralympic Games (29). Given the specificity of the group analyzed, the sample is considered adequate for the purposes of this study. Future research should include a larger number of VI judo matches from major international competitions (e.g., World Judo Championships) to increase the sample of actions, particularly regarding transitions to groundwork, as the current analysis was restricted to effective (scoring) transitions, not considering all non-scoring and non-progressed opportunities. This approach may limit the strength of performance-related conclusions and preventing full support for overall transition frequency, continuation rates into *ne-waza*, and opportunity-exploitation metrics considering each VI judo athletes, as suggested by Gutierrez-Santiago et al. (17).

From a practical perspective, the findings of this study can assist VI judo athletes, judo coaches, and strength and conditioning (S&C) coaches in developing targeted training strategies to optimize performance (30, 31) During the critical transition from standing to groundwork, various factors such as pace of transition, type of techniques, status, and scores constantly change throughout the match, and the present findings suggest that effective technical-tactical preparation should consider the higher frequency of successful transitions in J1 athletes, the predominance of *osae-komi-waza*, the exclusive use of *shime-waza* by J2 athletes, and the minimal influence of match moment or competition phase on transition success. These findings may offer preliminary insights into the performance characteristics of VI judo athletes, especially regarding transitions to groundwork techniques, which could be further investigated in subsequent studies.

## 5 Conclusion

The results suggest that J1 athletes exhibited a higher frequency of effective transitions from standing to groundwork combat compared to J2 athletes, with *osae-komi-waza* techniques leading to ippon more frequently for both groups. In contrast, *shime-waza* was more prevalent in J2 athletes compared to J1. No significant differences were observed for sex, weight category, competition phase, match moment, and status, indicating comparable strategies and execution across sport classes. These findings highlight technical-tactical profiles according to sport class and provide practical insights to guide training strategies and preparation for high-level competitions under the new classification system.

## Data Availability

The raw data supporting the conclusions of this article will be made available by the authors, without undue reservation.
